# Comparison of the fecal bacterial microbiota of healthy and diarrheic foals at two and four weeks of life

**DOI:** 10.1186/s12917-017-1064-x

**Published:** 2017-05-30

**Authors:** A. Schoster, H.R. Staempfli, L.G. Guardabassi, M. Jalali, J.S. Weese

**Affiliations:** 1 Equine Department, University of Zurich, Vetsuisse Faculty, Winterthurerstrasse 260, 8057 Zurich, Zurich Switzerland; 2 Department of Veterinary Disease Biology, University of Copenhagen, Faculty of Health and Medical Science, Stigbojlen 4, 1870, Copenhagen, Denmark; 3 Department of Clinical Studies, University of Guelph, Ontario Veterinary College, Guelph, N1G2W1 Canada; 4 Department of Biomedical Sciences, Ross University School of Veterinary Medicine, Basseterre, St Kitts and Nevis; 5 Department of Pathobiology, University of Guelph, Ontario Veterinary College, Guelph, N1G2W1 Canada

**Keywords:** Gastrointestinal microbiota, Metagenomic sequencing, Horse, Lachnospiraceae, Ruminococcaceae, Clostridiales

## Abstract

**Background:**

Diarrhea in foals affects up to 60% of foals during the first six months of life. The effect of diarrhea on the fecal bacterial microbiota in foals has not been investigated. Little is known on the fecal bacterial microbial richness and diversity of foals at a young age. The objective was to compare the fecal bacterial microbiota of healthy foals to foals with diarrhea at two and four weeks of life.

**Methods:**

Fecal samples were collected from foals (*n* = 20) at 1–14 (T1) and 15–28 (T2) days of age and analyzed using high throughput sequencing. Differences in relative abundance of bacterial taxa, alpha diversity and beta diversity indices were assessed between age-matched foals with diarrhea (*n* = 9) and healthy foals (*n* = 11), and between time points.

**Results:**

Differences in microbial community composition based on time point and health status were observed on all taxonomic levels. Of 117 enriched species in healthy foals at T2, 50 (48%) were Lachnospiraceae or Ruminococcaceae. The Chao richness index was increased in healthy foals at T2 compared to T1 (*p* = 0.02). Foals with diarrhea had a significantly lower richness index than non-diarrheic foals at T2 (*p* = 0.04). Diarrhea had an inconsistent effect, while time point had a consistent effect on microbial community structure.

**Conclusions:**

Preventative and therapeutic measures for diarrhea should focus on maintaining bacterial microbiota richness. Lachnospiraceae and Ruminococcaceae were underrepresented in foals with diarrhea. These should be evaluated further as potential therapeutic options.

**Electronic supplementary material:**

The online version of this article (doi:10.1186/s12917-017-1064-x) contains supplementary material, which is available to authorized users.

## Background

The bacterial microbiota plays a key role in human and animal health. Recent studies indicate that imbalances in the microbial communities and their function (dysbiosis) can be associated with diseases in the gastrointestinal tract and beyond [1]. Dysbiosis in horses has been associated with colitis [2], laminitis [3, 4], equine grass sickness [5], colic [6], and transient diarrhea in foals, known as foal heat diarrhea [7].

Age affects the composition of the gastrointestinal microbiota in humans and animals [8, 9]. The meconium of human infants already contains a distinct microbiota, and gastrointestinal colonization further develops upon contact with the maternal microbiota and environmental bacteria [10]. There have been limited studies in horses using techniques that can adequately evaluate the complex structure of gut microbial communities [11, 12]. A recent next generation sequencing based study reported that the fecal bacterial microbiota of foals is highly variable early in life, but reaches a relatively stable population by approximately 60 days of age [9]. However, the bacterial microbiota continues to develop throughout adolescence. Differences in community structure, evidenced by how many and which species are present as well as their relative abundance, are still present in foals after 60 days of age compared to adult horse [9].

Foal diarrhea is common and important, reportedly affecting 60% of foals during their first six months of life [13]. A single causative agent is often not identified [14]. Acute diarrhea is associated with significant changes in the fecal bacterial microbiota of adult horses. Marked differences in composition of the fecal bacterial microbiota over all taxonomic levels and decreases in bacterial diversity, evenness and richness have been described [2, 15]. Despite the high prevalence of foal diarrhea, there is currently little knowledge about the fecal bacterial microbiota of foals affected by diarrhea. A culture-based study showed that changes in the fecal bacterial microbiota over time did not differ between foals with diarrhea and healthy foals [7]; however, culture-based methods have limited ability to appreciate the complexity of the bacterial microbiota [16].

The objectives of this study were to describe differences between foals with and without diarrhea at two and four weeks of life.

## Methods

The University of Guelph Animal Care Committee (AUP 1455) approved this study.

### Animals and study protocol

Foals born on one Standardbred and seven Thoroughbred farms in southern Ontario, Canada, were studied. Mares and foals were turned out on pasture during the day and stabled overnight if weather permitted. Mares were fed coastal hay ad libitum and were allowed to graze on pasture. Type and amount of supplemental feed (grain, minerals etc.) differed between farms. Probiotics were not administered to mares or foals. Fecal swabs and medical records were collected from foals as part of a separate study [14]. Experienced farm personnel recorded fecal consistency daily for 28 days on a standardized medical record sheet, which was provided. Foals were included in this study if they were born by natural delivery, were clinically normal at the time of enrollment (24 h after birth), had two fecal samples collected sequentially and had a complete medical record sheet. Exclusion criteria were presence of severe gastrointestinal disease or illness by 24 h of life as well as administration of antimicrobials, anti-inflammatories, probiotics or Biosponge^1^ at any point during the study period. Sampling days were dependent on farm management, which resulted in samples being taken during two time periods 1–14 (T1) and 15–28 (T2) days of age. It was determined based on the medical record whether a foal had diarrhea at the time of sampling. Fecal samples from foals with diarrhea were collected within two days after diarrhea occurrence. Foals included in the healthy group did not have diarrhea on any day throughout the study period. The samples were stored at 4 °C for a maximum of two weeks before being transported to the laboratory where all were frozen at −80 °C until DNA extraction.

### DNA extraction, 16S rRNA gene amplification, purification and sequencing

Fecal swabs were reconstituted in 1 mL of phosphate buffered saline. The suspension was centrifuged at 9800 g for 4 min and DNA was extracted from the pellet using a commercial kit (E.Z.N.A. Stool DNA Kit^2^) according to manufacturer’s recommendations. Adequate DNA quality and quantity were assessed by spectrophotometry (NanoDrop^3^).

Amplification of the V4 region of the 16S rRNA gene, purification and sequencing were performed as previously described [17]. Briefly, primers targeting the V4 region of the 16S rRNA gene were designed with overhanging adapters for annealing to the Illumina index primers in the second PCR step. PCR products were purified and Illumina index primers were attached during the second PCR step. PCR products were purified and evaluated by gel-electrophoresis in 1.5% agarose gel. The samples were sequenced at the University of Guelph’s Advanced Analysis Centre using an Illumina MiSeq (Illumina RTA v1.17.28; MCS v2.2).

### Bioinformatics and statistics

Distribution of sex, breed and farm was compared between foals with and without diarrhea using Fisher’s exact test. The age at the time of sampling between foal with and without diarrhea at a given time point was compared using the Mann-Whitney U test as data was not normally distributed.

The open-source software package, MOTHUR (v1.33.0) was used to process the sequences [18]. Paired end reads were aligned and then sequences were aligned with the SILVA 16S rRNA gene reference database (www.arb-silva.de) [19]. Sequences with ambiguous base calls, inappropriate length (>244 base pairs (bp) or <239 bp), runs of homopolymers of >8 bp, and sequences corresponding to chloroplasts, mitochondria, Archaea and Eukaryotes were removed. Chimeras were identified using uchime [20] and removed. The remaining sequences were classified using the ribosomal database project (RDP) classifier (http://rdp.cme.msu.edu/index.jsp). Subsampling was performed to normalize sequence numbers for further comparison. This consisted of random selection of a number of sequences from each sample that corresponded to the lowest sequence abundance of all samples. Completeness of sampling effort was assessed visually using rarefaction curves.

Alpha diversity was described using the Chao richness, Shannon’s evenness and inverse Simpson’s index. The two main factors to quantify biological diversity are richness and evenness. Richness (represented here by the Chao richness index) describes the number of species per sample. Evenness (represented by the Shannon evenness index) describes the relative abundance of the different species making up the richness. A community dominated by one or two species is considered less diverse than a community in which several species have a similar abundance. Simpsons diversity index is a measure of diversity which takes into account both richness and evenness.

Only bacterial taxa accounting for more than 1 % of the total were used for statistical analysis. Data were determined to be non-parametric based on examination of quantile plots and Shapiro-Wilk testing. Relative abundances and alpha diversity indices were compared between healthy and diarrheic foals and between age groups in healthy foals using the Wilcoxon test. Observed species richness was compared between healthy foals and foals with diarrhea and between age groups in healthy foals using the Wilcoxon matched pairs signed rank test. False discovery rate (FDR) adjustments using the Benjamin Hochberg procedure were performed for comparisons of relative abundance of taxa between age groups and between foals with and without diarrhea.

Dendograms based on community overlap (classical Jaccard index) and structure (Yue&Clayton index of dissimilarity) were created and visualized by FigTree v1.4.0 (http://tree.bio.ed.ac.uk/). Community overlap and structure were compared between groups by parsimony test, analysis of molecular variance (AMOVA), and analysis of similarities (ANOSIM) applied to the Jaccard and Yue&Clayton data, respectively. The Jaccard index is a measure of the number of species shared between two samples (richness), while the Yue&Clayton index takes into account relative abundance of each species. Dissimilarity was also visualized using principal coordinate analysis (PCoA). Linear discriminant analysis effect size (LEfSe) was performed to identify differentially abundant operational taxonomic units (OTUs) with 97% sequence similarity between groups. [21] A *p*-value of <0.05 was considered significant for all comparisons. A commercial program was used for all statistical analyses (JMP Statistical discoveries, Version 11).

## Results

### Demographic data of animals

Twenty foals, 2/20 (10%) Standardbreds and 18/20 Thoroughbreds (90%) were included. Thirteen foals (65%) were male and 7 (35%) were female. Nine of 20 (45%) foals developed diarrhea at some point during the study period (Table 1). Significantly more females developed diarrhea compared to males (*p* = 0.02). Breed and farm were not significantly different between foals with and without diarrhea (*p* = 0.19 and *p* = 0.23, respectively.)

**Table 1:** Population data on foals studied

Foal	Breed	Sex	Farm	Diarrhea	Age at T1 (days)	Age at T2 (days)	Diarrhea at T1	Diarrhea at T2
1	TB	M	1	No	8	23		
2	TB	M	2	No	4	22		
3	TB	M	1	No	14	26		
4	TB	F	2	Yes	7	17	No	Yes
5	TB	F	1	Yes	7	14	Yes	Yes
6	TB	F	3	Yes	1	15	Yes	No
7	TB	M	4	No	9	25		
8	TB	M	5	No	8	22		
9	TB	F	2	Yes	11	18	Yes	No
10	TB	M	6	No	5	25		
11	TB	M	2	No	10	28		
12	TB	M	7	Yes	7	21	No	Yes
13	TB	M	1	No	5	15		
14	TB	M	1	No	14	28		
15	SB	F	8	Yes	5	19	Yes	No
16	TB	F	6	No	9	20		
17	TB	M	7	No	10	25		
18	TB	M	7	Yes	8	22	No	Yes
19	SB	F	8	Yes	12	26	No	Yes
20	TB	M	2	Yes	5	15	No	Yes

*SB* Standardbred, *TB* Thoroughbred, *F* Female, *M* Male, T1: 1–14 days of age, T2: 15–28 days of age

### Sequencing quality data

The DNA concentration after extraction ranged from 5 to 50 ng/μL. Final concentration of PCR products before sequencing averaged approximately 30 ng/μL. A total of 4,914,371 V4 16S RNA gene sequences passed all quality control filters. Subsampling was performed based on 29,980 sequences per sample. Sequencing depth was deemed adequate based on rarefaction curves (Additional file 1).

### Microbial community composition

**Fig. 1:**
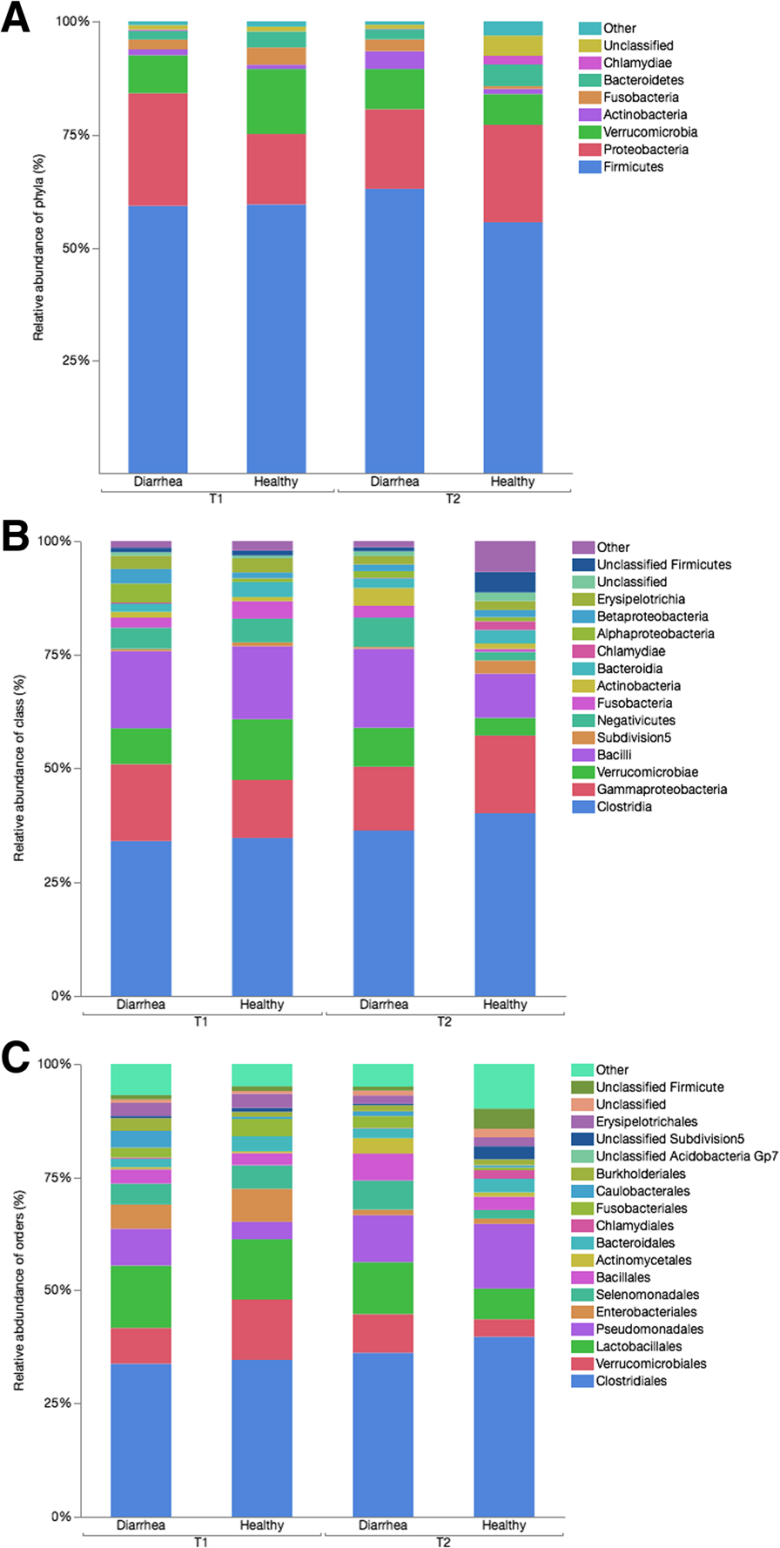
Relative abundance of predominant phyla (**a**), classes (**b**), and orders (**c**), in healthy foals (*n* = 11) and foals with diarrhea (*n* = 4 at T1, *n* = 3 at T2). Other: Bacterial taxa with ≤1% abundance, Unclassified: Sequences which could not be calssified, T1: 1–14 days of age, T2: 15–28 days of age

Twenty-nine different phyla, 71 classes, 129 orders were identified. Seven phyla, 14 classes, and 16 orders had a mean relative abundance of more than 1% (Fig. 1). Of 250 families identified, 19 had a mean relative abundance of >1%. The most abundant family was Ruminococcaceae (13.1%), followed by Lachnospiraceae (12.7%), Verrucomicrobiaceae (8.4%), Moraxellaceae (5.2%), Carnobacteriaceae (4.3%), Pseudomonaceae (3.9%), Enterobacteriaceae (3.8%), Veillonellaceae (3.6%), Lactobacillaceae (3.1%), Erysipelotrichaeceae (2.4%), Fusobacteriaceae (2.3%), Clostridiaceae I (1.9%), Bacillaceae (1.7%), Bacteroidaceae (1.5%), unclassified Subdivsion 5 (1.2%), unclassified Clostridales XI (1.1%), Caulobacteriaceae (1.3%), Enterococcaceae (1.3%) and Planococcaceae (1.2%).

### Fecal microbial composition of healthy foals at two and four weeks of age

**Fig. 2:**
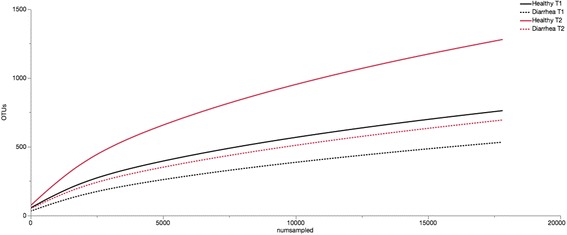
Rarefication analysis (number of observed species) of the 16S rRNA gene sequences obtained from foal fecal samples, T1: 1–14 days of age, T2: 15–28 days of age. Each line represents the average of a group: healthy foals (*n* = 11) and foals with diarrhea (*n* = 4 at T1 and *n* = 3 each at T2). The analysis was performed on a selected subset of 17,800 sequences (lowest sequence number of all samples)

**Fig. 3:**
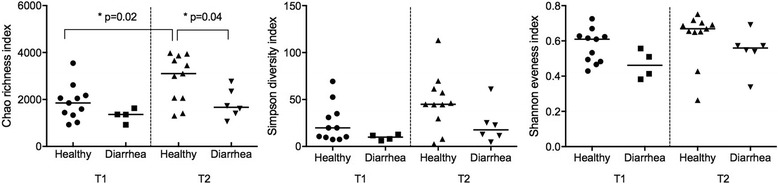
Median alpha diversity indices of the fecal bacterial microbiota of healthy foals (*n* = 11) and foals with diarrhea (*n* = 4 at T1 and *n* = 6 at T2). T1: 1–14 days of age, T2: 15–28 days of age. Horizontal line represents the median

For comparisons between the two time points, only samples from healthy foals (*n* = 11) that did not develop diarrhea throughout the study were analyzed. There were significant differences at all taxonomic levels (Table 2). The number of observed species was significantly lower in foals at T1 compared to T2 (*p* < 0.001, Fig. 2). The estimated richness (Chao index) significantly increased over time (*p* = 0.02). The other alpha diversity indices (Simpson diversity index Shannon evenness index) were not significantly different between T1 and T2 (*p* = 0.09 and *p* = 0.08, respectively, Fig. 3).

**Table 2:** Relative abundance of significantly different taxa over time in the fecal bacterial microbiota of healthy foals (*n* = 11)

	Median relative abundance % (CI %)		*p*-value	FDR adjusted *p*-value
	T1	T2		
Phyla				
Chlamydiae	0.001 (0–0.004)	0.2 (0.007–0.9)	0.005	0.04
Classes				
Chlamydiae	0.001 (0–0.004)	0.2 (0.007–0.9)	0.005	0.04
Verrucomicrobia Subdivision 5	0.2 (0.01–0.6)	3.2 (1.4–4.1)	0.005	0.04
Orders				
Chlamydiales	0.001 (0–0.004)	0.2 (0.007–0.9)	0.005	0.046
Unclassified Verrucomicrobia Subdivision 5	0.2 (0.01–0.6)	3.2 (1.4–4.1)	0.005	0.046
Families				
Unclassified Verrucomicrobia Subdivision 5	0.2 (0.01–0.6)	3.2 (1.4–4.1)	0.005	0.12
Clostridiaceae XI	0.01 (0.003–0.04)	0.2 (0.05–0.9)	0.02	0.44
Unclassified Clostridiales	1.9 (0.5–4.7)	9.2 (3.3–16.9)	0.02	0.14

FDR: False discovery rate adjusted (Benjamin Hochberg procedure)

T1: 1–14 days of age, T2: 15–28 days of age

**Fig. 4:**
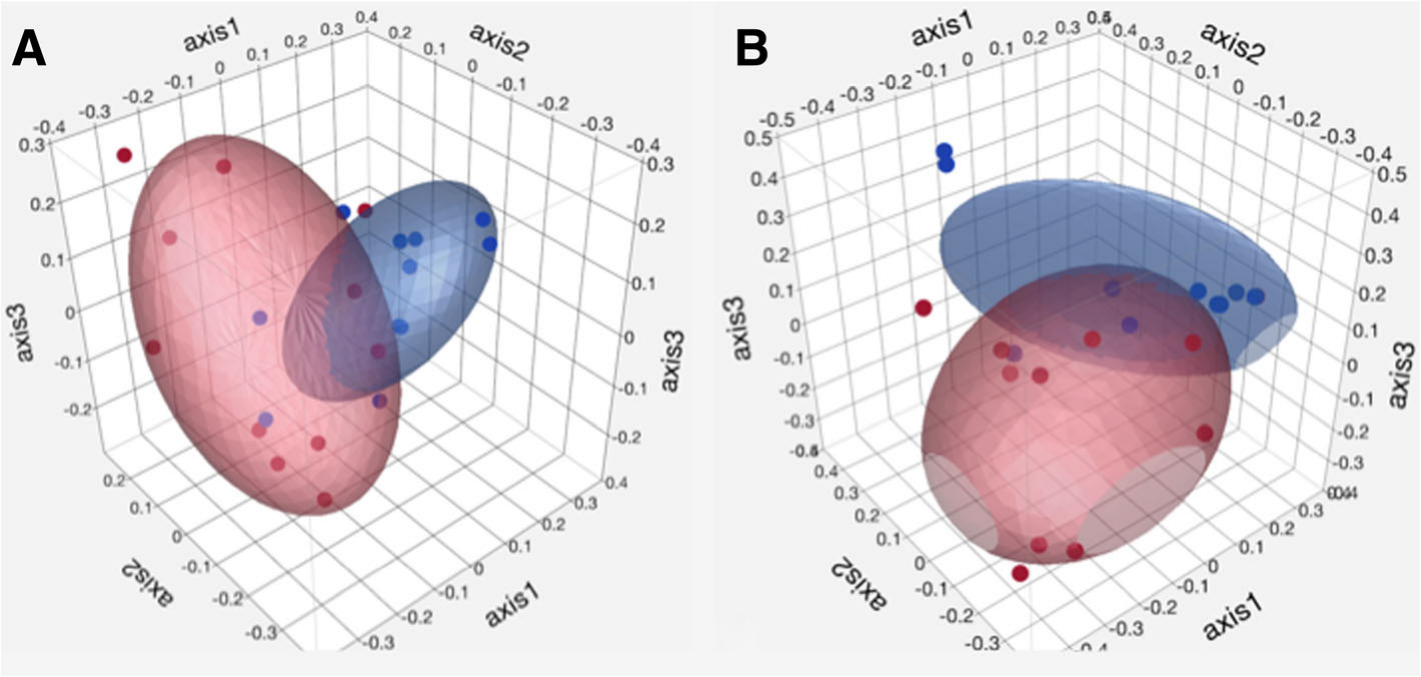
Principal coordinate analysis of the fecal bacterial microbiota of healthy foals (*n* = 11) at different time points. Principal coordinate analysis based on the Jaccard (**a**) and Yue&Clayton (**b**) index. Red: T1: 1–14 days of age, Blue: T2: 15–28 days of age. Ellipsoid coverage: 50%

Two distinct clusters (T1 and T2) were evident visually with PCOA based on the Jaccard and Yue&Clayton indices (Fig. 4). Samples from foals in the same age group tended to cluster together. Significant differences between T1 and T2 were identified by parsimony test for both indices (Yue&Clayton *p* = 0.04, Jaccard *p* = 0.006). Significant differences were also observed with AMOVA (Jaccard *p* = 0.003, Yue&Clayton *p* = 0.001) and ANOSIM (Jaccard *p* = 0.002, Yue&Clayton *p* = 0.001).

### Fecal microbial composition between healthy and diarrheic foals

To assess the influence of diarrhea on microbial community diversity, foals with diarrhea at the time of fecal sample collection were compared to age matched healthy foals (*n* = 11). A total of nine foals developed diarrhea throughout the study period. At T1 four foals had diarrhea, at T2 six foals had diarrhea. There was no statistically significant age difference between foals with and without diarrhea at T1 or T2 (*p* = 0.29 and *p* = 0.07, respectively).

Relative abundance was not significantly different after FDR adjustment on all taxonomic levels at either time point (Table 3). At T1, eight taxa were identified as enriched using LEfSe in the healthy group (*Actinobacillus, Pseudoflavonifractor*, unclassified Lachnospiraceae, *Lactobacillus*, unclassified Clostridiales, *Subdoligranulum, Streptococcus,* unclassified Verrucomicrobiaceae). Three taxa were enriched in the diarrhea group at T1 (*Aerococcus*, unclassified Erysipelotrichaceae, *Blautia*). At T2, 117 taxa were enriched in the healthy group, 50 (48%) of which were members of the families Lachnospiraceae or Ruminococcaceae (full list, *P*-values and LDA values are shown in Additional file 2).

**Table 3:** Relative abundance of significantly different taxa in the fecal bacterial microbiota of foals with (*n* = 4 at T1, *n* = 6 at T2) and without (*n* = 11) diarrhea

	Age group	Median relative abundance % (CI %)		*p*-value	FDR adjusted *p*-value
		Healthy	Diarrhea		
Phylum					
Chlamydiae	T2	0.2 (0.01–0.9)	<0.001(0- < 0.001)	0.02	0.08
Class					
Chlamydiae	T2	0.2 (0.01–0.9)	<0.001(0- < 0.001)	0.02	0.11
Verrucomicrobia Subdivision 5	T2	3.2 (1.4–4.1)	0.05 (<0.011–0.3)	0.004	0.06
Order					
Chlamydiales	T2	0.2 (0.01–0.9)	<0.001(0- < 0.001)	0.02	0.14
Unclassified Verrucomicrobial Subdivison 5	T2	3.2 (1.4–4.1)	0.05 (<0.011–0.3)	0.004	0.08
Family					
Ruminococcaceae	T1	18.2 (0.6–8.5)	1.8 (4.5–23.3)	0.04	1
Clostridiaceae	T2	0.7 (0.3–3)	0.2 0.1–0.4)	0.04	0.35

FDR: False discovery rate adjusted (Benjamin Hochberg procedure)

T1: 1–14 days of age, T2: 15–28 days of age

The number of observed species was significantly lower in foals with diarrhea at T1 and T2 (both *p* < 0.001, Fig. 2). There was also a significant decrease in estimated richness (Chao index) in foals with diarrhea compared to healthy foals at T2 (*p* = 0.04, Fig. 3); however, not at T1 (*p* = 0.15). No other differences in alpha diversity were identified (all *p* > 0.61).

**Fig. 5:**
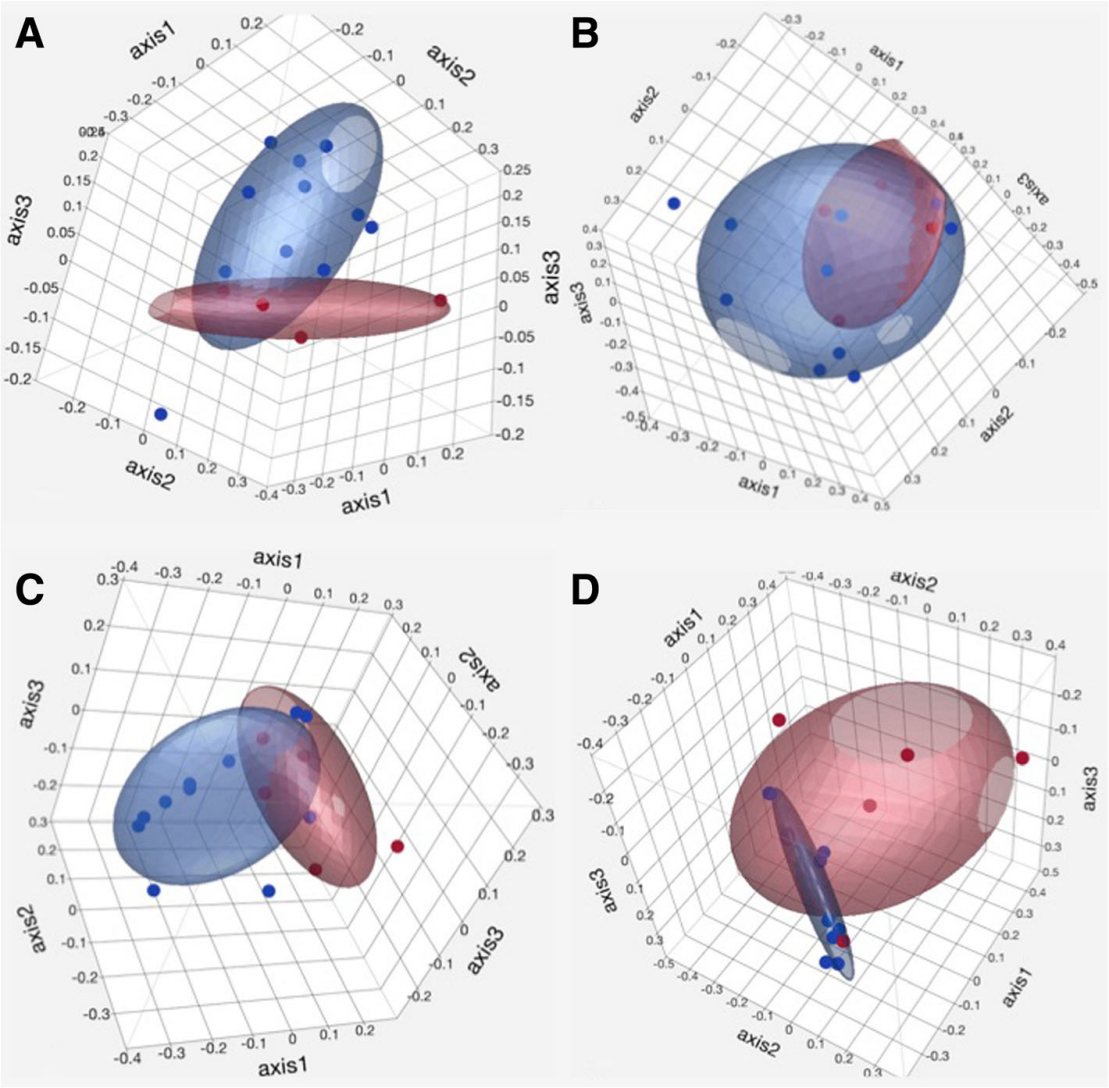
Principal coordinate analysis of the fecal bacterial microbiota of healthy foals and foals with diarrhea. Principal coordinate analysis based on the Jaccard index (panel **a** for T1 and panel **c** for T2) and the Yue&Clayton index (panel **b** for T1 and panel **d** for T2). T1: 1–14 days of age, T2: 15–28 days of age. Blue: healthy foals (*n* = 11), red: foals with diarrhea (*n* = 4 at T1 and *n* = 6 at T2, red). Ellipsoid coverage: 50%

Distinct clusters (healthy foals vs. foals with diarrhea) were not visually evident on Jaccard and Yue&Clayton PcOA (Fig. 5) and a statistical difference in community structure was not consistent across statistical tests (Table 4).

**Table 4:** Difference in bacterial microbial composition (Jaccard index) and structure (Yue&Clayton index) of the fecal bacterial microbiota of foals with (*n* = 4 at T1, *n* = 6 at T2) and without (*n* = 11) diarrhea

	T1		T2	
	Jaccard index *p*-value	Yue&Clayton index *p*-value	Jaccard index *p*-value	Yue&Clayton index *p*-value
Parsimony	0.1	1	0.61	0.07
AMOVA	0.42	0.17	0.01	0.17
ANOSIM	0.21	0.04	0.009	0.009

T1: 1–14 days of age, T2: 15–28 days of age

## Discussion

The bacterial microbiota of foals is assumed to play an important role in diarrhea, and early colonization and development is a dynamic process. In this study, significant impacts of both age and diarrhea were noted, with the most evident influential factor being age.

Knowledge of the bacterial microbiota in foals is still in its infancy. Initial culture-based studies tended to focus on a narrow spectrum of the fecal bacterial microbiota, such as lactobacilli, enterococci and clostridia [22]. Subsequent culture-independent studies have provided much more insight into the rapid development of the bacterial microbiota. Foals develop a rich and diverse bacterial microbiota early in life, and it has been previously reported that the foal’s fecal bacterial microbiota resembles that of adult horses by 60 days of age. [9]. Here, changes over time in microbial composition were less evident than reported in the above study, something that is unsurprising because of the short study period. Regardless, there were many significant differences in the fecal bacterial microbiota even over this relatively short timeframe, highlighting the dynamic nature of the microbiota. Similar to the only comparable study of the fecal bacterial microbiota of foals, the most abundant bacterial phyla here were Firmicutes, Verrucomicrobia and Proteobacteria [9]. The most abundant families were members of the Clostridia class, Ruminococcaceae and Lachnospiraceae which is also in agreement with a prior study [9].

The field nature of the study and farm management factors made it impossible to collect fecal samples on specified days in this study. This resulted in a wide age range (up to 14 days) in each group. Further, the period between the two samples was not consistent between foals. This complicates interpretation and analysis of the data, particularly trying to determine the effect of age on the microbiota. Nevertheless, further data was added to the growing knowledge on the development of the bacterial microbiota, and the age factor has to be considered when designing and interpreting microbial studies.

In a study in adult horses with colitis changes on all taxonomic levels were identified [2]. Changes in members of higher taxonomic levels, such as phylum, order and class, presumably indicate a more profound change of the bacterial microbiota and are more likely to result in functional differences compared to changes in lower taxonomic levels. In our study none of the changes was significant after FDR adjustment. However, some differences in fecal bacterial microbiota composition and structure were evident.

An apparent underrepresentation of Lachnospiraceae and Ruminococcaceae, the two most abundant families, were seen in foals with diarrhea in this study. Lachnospiraceae and Ruminococcaceae, members of the Clostridia class, have been implicated as having important roles in gastrointestinal health in numerous animal species. In humans and animals with gastrointestinal disease these species were consistently under represented, independent of the cause of gastrointestinal disease [23–27]. Clostridia are major producers of short chain fatty acids (SCFA), which are important for intestinal and general health [28, 29]. *Faecalibacterium*, a member of the Ruminococcaceae, has also been shown to produce anti-inflammatory peptides in-vitro [30] and has been shown to decrease diarrhea incidence and mortality in calves [31]. A decrease of these bacterial families in horses with colitis and colic as well as horses exposed to stress factors such as fasting, transport and anesthesia has also been shown [2, 6, 32] and a decrease in some of these members has been identified prior to the onset of colic in post-partum mares [6]. The underrepresentation of members of these families, such as *Blautia*, *Faecalibacterium* and *Ruminococcus* among others, in foals with diarrhea further supports the potential importance of these bacterial families.

Richness was lower in foals with diarrhea compared to healthy foals. Gut bacteria are involved in many homeostatic processes through metabolite production and communication with the host’s immune system. While optimal levels of richness are not known, a diverse bacterial microbiota is thought to be of use as it may be more adept at fulfilling a wide range of necessary functions and be more resilient in the face of changes (e.g. nutritional change, stress). Loss of bacterial diversity and richness has been demonstrated in acute and chronic gastrointestinal enteropathies in humans and animals, including wide-ranging disorders such as Crohn’s disease [33], inflammatory bowel disease [30] and acute enteric infections [25] in humans, acute diarrhea [27] and inflammatory bowel disease [34] in dogs and acute colitis in adult horses [15].

The complexity of the fecal bacterial microbiota, and the finding that bacterial alpha diversity is decreased in foals with diarrhea raises questions about common approaches to prevention or treatment of diarrhea. Metronidazole is commonly used to treat diarrhea in horses and sometimes foals [35]. Many members of the Clostridia class are susceptible to this drug [36]. Therefore, treatment could potentially inhibit the beneficial components of the fecal bacterial microbiota. Probiotics are also commonly used, although to date several studies have not shown any clear benefit for treatment or prevention of diarrhea in foals [12, 14, 37, 38]. One potential reason could be that the organisms found in most commercial probiotics (e.g. enterococci, lactobacilli) are not those found to be important members of the healthy bacterial microbiota in several studies including our study [2, 39]. This raises questions about the potential efficacy of conventional probiotic organisms for the treatment or prevention of diarrhea, especially when they have to compete in such as rich and abundant existing bacterial microbiota.

Antimicrobial treated foals were excluded as it has been shown that antimicrobial administration causes profound and sustained changes in the equine fecal bacterial microbiota [40]. Foals were included in the study irrespective of the cause of diarrhea. It is unlikely that this influenced the results, as it has been shown in humans that intestinal microbial communities become affected in a similar way, irrespective of the pathogen type and age [25]. Severity, duration and frequency of diarrhea were not taken into account during analysis of these data. Given the small number of foals and large variation in diarrhea duration, this was no feasible.

Factors other than age can impact the fecal bacterial microbiota and could not be accounted for. Foals from seven farms over a period of two breeding seasons were enrolled to acquire a sufficient number of foals [14], creating some potential for farm- and year-based variation. High starch diets and feed changes have been shown to have a significant impact on the microbial communities [41]. Foals less than four weeks of age are not commonly fed large amounts of high starch feed; therefore, significant bias based on farm is unlikely. There are no studies evaluating the effect of other management practices, it is therefore difficult to assess how, and to what extent the factor farm could have influenced these results. Exact feeding and management practices were not available for each farm but should be collected or unified in future studies to better assess the effect of diarrhea with confounding factors.

More females than males were affected by diarrhea. Females could have inherent differences in fecal bacterial microbiota composition compared to males, which could influence results of the effect of diarrhea. This has not been studied in horses, however has been investigated in human medicine, and differences in microbial composition between man and women are present [42]. It cannot be predicted to what extent and in which way this would have influenced our results.

Given that age has an effect on microbial composition, alpha- and beta diversity, we tried to control for this factor when sampling the foals; however, foals with diarrhea had fecal samples taken at an earlier age than healthy foals at time point two. This could have also influenced the results and makes it difficult to interpret the exact effect of diarrhea versus a lingering effect of age in these foals.

Another limitation of this study was the differences in storage conditions for some of the samples. Some samples were stored at 4 °C for up to 2 weeks before being frozen at −80 °C. There is no data on how different storage conditions affect the equine fecal bacterial microbiota; however, it has been shown in dogs and cats that storage at 4 °C for up to 14 days has limited effects on the fecal bacterial microbiota composition. Changes in limited groups occurred but there were no differences in richness, diversity, evenness or community structure [43]. It is likely that this is similar for the equine fecal bacterial microbiota. Storage conditions should not have significantly affected the results of our study, particularly in regards to richness and diversity on the species level.

## Conclusions

Bacterial richness is decreased in foals with diarrhea, suggesting that potential preventative and therapeutic measures should focus on providing and maintaining a diverse and rich bacterial microbiota in addition to defining and modifying the key bacterial species involved in gastrointestinal health, such as members of the Lachnospiraceae and Ruminococcaceae.

## Additional files

Additional file 1: Rarefecation curve. Rarefaction curves of V4 16S rRNA gene sequences from fecal samples from neonatal foals (*n* = 20) sampled at 1–14 and 15–28 of age. (TIFF 17294 kb)

Additional file 2: LEfSe Results. Title of date: Analysis from LEfSe analysis. Species significantly enriched in the fecal microbiota of healthy foals (*n* = 11) and foals with diarrhea (*n* = 4 at T1 and *n* = 6 at T2) sampled over time determined by linear discriminant analysis effect size. (LEfSe) (PDF 87 kb)

## Abbreviations

AMOVA: Analysis of molecular variance
ANOSIM: Analysis of similarities
bp: Base pairs
FDR: False discovery rate
LEfSe: Linear discriminant analysis effect size
OUT: Operational taxonomic unit
PcOA: Principal coordinate analysis
RDP: Ribosomal database project

## Footnotes

1 Bio-Sponge, Platinum Peformance, Buellton, CA.

2 E.Z.N.A. Stool DNA Kit, Omega Bio-Tek Inc, GA, USA

3 NanoDrop, Roche, ON, Canada
