# Supplementary figures and images for: Comparison of the fecal bacterial microbiota of healthy and diarrheic foals at two and four weeks of life
Source: BMC Vet Res. 2017 May 30;13:144. doi: 10.1186/s12917-017-1064-x (PMC5450145; doi:10.1186/s12917-017-1064-x)

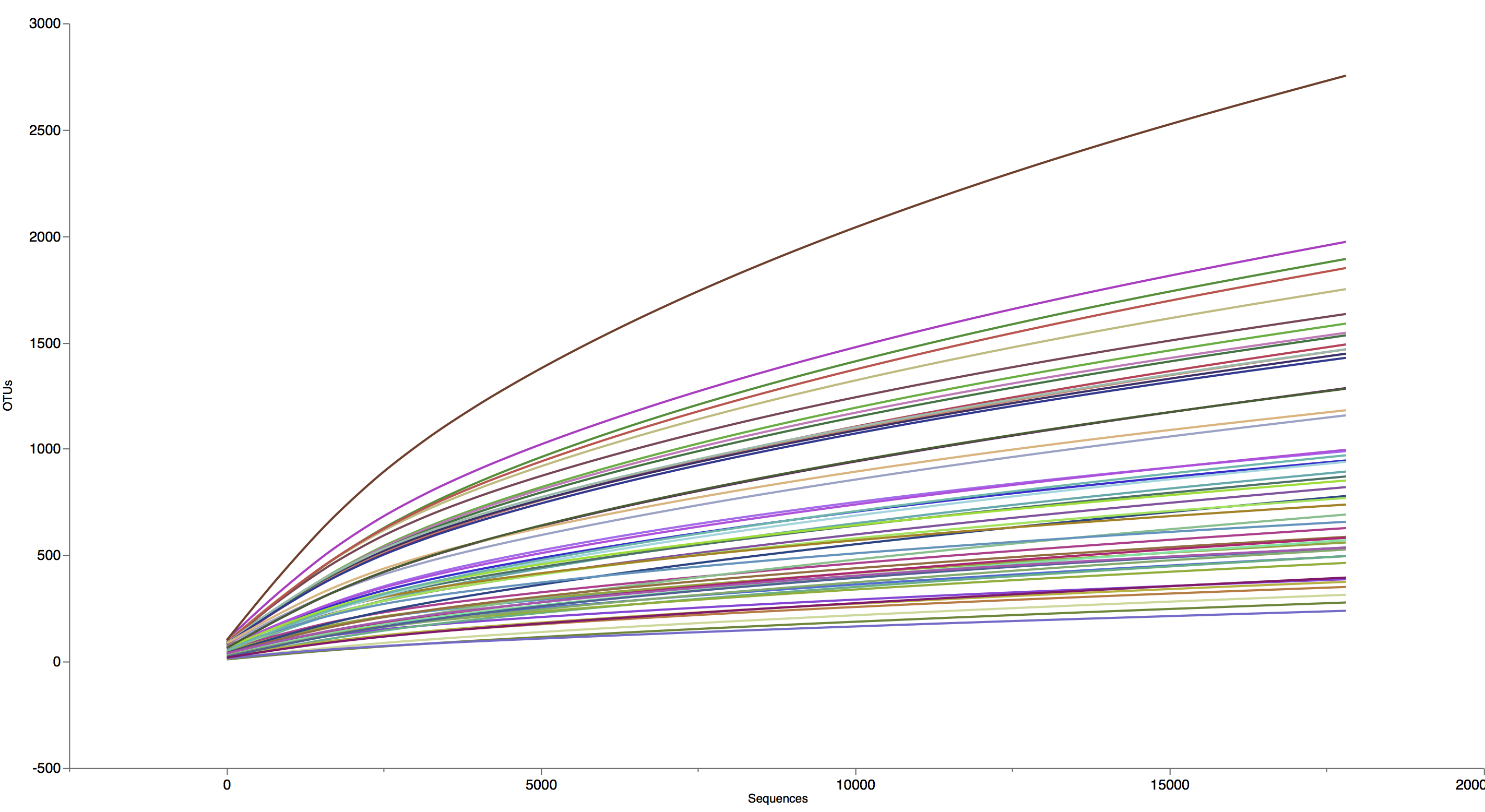

Supplement: Supplementary file 1 — Rarefecation curve. Rarefaction curves of V4 16S rRNA gene sequences from fecal samples from neonatal foals (n = 20) sampled at 1–14 and 15–28 of age. (TIFF 17294 kb) [file 12917_2017_1064_MOESM1_ESM.tiff]
