# Supplementary material for: Comparison of the fecal bacterial microbiota of healthy and diarrheic foals at two and four weeks of life
Source: BMC Vet Res. 2017 May 30;13:144. doi: 10.1186/s12917-017-1064-x (PMC5450145; doi:10.1186/s12917-017-1064-x)
Supplement: Supplementary file 2 — LEfSe Results. Title of date: Analysis from LEfSe analysis. Species significantly enriched in the fecal microbiota of healthy foals (n = 11) and foals with diarrhea (n = 4 at T1 and n = 6 at T2) sampled over time determined by linear discriminant analysis effect size. (LEfSe) (PDF 87 kb) [file 12917_2017_1064_MOESM2_ESM.pdf]

Additional file 2 Results from LEfSe analysis

Species significantly enriched in the fecal microbiota of healthy foals (n=11) and foals with diarrhea (n=4 at T1 and n=6 at T2) sampled over time determined by linear discriminant analysis effect size (LEfSe)

OTU	Family	Health	LDA	p-value
**T1**				
Actinobacillus	Pasteurellaceae	Healthy	3.48	0.05
Pseudoflavonifractor	Ruminococcaceae	Healthy	3.39	0.03
Unclassified Lachnospiraceae	Lachnospiraceae	Healthy	3.37	0.01
Lactobacillus	Lactobacillaceae	Healthy	3.25	0.04
Unclassified Clostridiales	Unclassified Clostridiales	Healthy	3.19	0.03
Subdoligranulum	Ruminococacaeae	Healthy	3.15	0.04
Streptococcus	Streptococcaceae	Healthy	2.72	0.05
Unclassified Verrucomicrobiaceae	Verrucomicrobiaceae	Healthy	2.21	0.05
Aerococcus	Lactobacillaceae	Diarrhea	3.05	0.05
Unclassified Erysopelatrichaceae	Erysopelotrichaceae	Diarrhea	2.26	0.02
Blautia	Lachnopsiraceae	Diarrhea	2.12	0.02
**T2**				
Unclassified Ruminococcaceae	Ruminococcaceae	Healthy	4.08	0.03
Chlamydophila	Chlamydiaceae	Healthy	3.98	0.04
Unclassified Subdivison 5	Unclassified Subdivision 5	Healthy	3.76	0.00
Sarcina	Clostridiaceae	Healthy	3.75	0.03
Unclassified	Unclassified	Healthy	3.59	0.00
Sporobacter	Ruminococcaceae	Healthy	3.37	0.02
Unclassified Clostridiales	Unclassified Clostridiales	Healthy	3.26	0.03
Oscillibacter	Ruminococcaceae	Healthy	3.18	0.03
Unclassified Subdivison 5	Unclassified Subdivision 5	Healthy	3.16	0.00
Acidaminococcus	Acidaminococcaceae	Healthy	3.14	0.02
Unclassified Ruminococcaceae	Ruminococcaceae	Healthy	3.12	0.04
Unclassified Ruminococcaceae	Ruminococcaceae	Healthy	3.11	0.02
Treponema	Spirochetaceae	Healthy	3.10	0.01
Unclassified Subdivision 5	Unclassified Subdivision 5	Healthy	3.06	0.00
Unclassified Lachnospiraceae	Lachnospiraceae	Healthy	3.05	0.03
Acidaminococcus	Acidaminococcaceae	Healthy	3.03	0.03
Unclassified Ruminococcaceae	Ruminococcaceae	Healthy	4.08	0.03
Chlamydophila	Chlamydiaceae	Healthy	3.98	0.04
Unclassified Subdivison 5	Unclassified Subdivision 5	Healthy	3.76	0.00
Sarcina	Clostridiaceae	Healthy	3.75	0.03
Unclassified	Unclassified	Healthy	3.59	0.00
Sporobacter	Ruminococcaceae	Healthy	3.37	0.02
Unclassified Clostridiales	Unclassified Clostridiales	Healthy	3.26	0.03
Oscillibacter	Ruminococcaceae	Healthy	3.18	0.03
Unclassified Subdivison 5	Unclassified Subdivision 5	Healthy	3.16	0.00

Acidaminococcus	Acidaminococcaceae	Healthy	3.14	0.02
Unclassified Ruminococcaceae	Ruminococcaceae	Healthy	3.12	0.04
Unclassified Ruminococcaceae	Ruminococcaceae	Healthy	3.11	0.02
Treponema	Spirochetaceae	Healthy	3.10	0.01
Unclassified Subdivision 5	Unclassified Subdivision 5	Healthy	3.06	0.00
Unclassified Lachnospiraceae	Lachnospiraceae	Healthy	3.05	0.03
Acidaminococcus	Acidaminococcaceae	Healthy	3.03	0.03
Treponema	Spirochetaceae	Healthy	3.00	0.03
Unclassified Sphingobacteriales	Unclassified Sphingobacteriales	Healthy	2.97	0.03
Unclassified Ruminococcaceae	Ruminococcaceae	Healthy	2.96	0.04
Unclassified Betaproteobacteria	Unclassified Betaproteobacteria	Healthy	2.90	0.03
Unclassified Erysopelotrichaceae	Erysopelatrichaceae	Healthy	2.88	0.03
Unclassified Lachnospiraceae	Lachnospiraceae	Healthy	2.87	0.02
Unclassified Ruminococcaceae	Ruminococcaceae	Healthy	2.85	0.03
Unclassified Lachnospiraceae	Lachnospiraceae	Healthy	2.81	0.03
Clostridium IV	Ruminococcaceae	Healthy	2.77	0.05
Unclassified Subdivision 5	Unclassified Subdivision 5	Healthy	2.77	0.01
Saccharofermentans	Ruminococcaceae	Healthy	2.77	0.02
Unclassified Lachnospiraceae	Lachnospiraceae	Healthy	2.75	0.00
Acetivibrio	Ruminococcaceae	Healthy	2.69	0.01
Mogibacterium	Unclassified Clostridiales	Healthy	2.67	0.01
Unclassified Lachnospiraceae	Lachnospiraceae	Healthy	2.62	0.02
Unclassified Lachnospiraceae	Lachnospiraceae	Healthy	2.61	0.03
Unclassified Erysopelotrichaceae	Erysopelotrichaceae	Healthy	2.60	0.02
Unclassified Clostridiales	Unclassified Clostridiales	Healthy	2.59	0.03
Lactonifactor	Lachnospiraceae	Healthy	2.58	0.04
Unclassified Subdivision 5	Unclassified Subdivision 5	Healthy	2.57	0.01
Holdemania	Erysopelotrichaceae	Healthy	2.57	0.01
Unclassified Clostridiales	Unclassified Clostridiales	Healthy	2.54	0.03
Paraprevotella	Prevotellaceae	Healthy	2.54	0.04
Unclassified Lachnospiraceae	Lachnospiraceae	Healthy	2.53	0.02
Unclassified Firmicutes	Unclassified Firmicutes	Healthy	2.51	0.04
Unclassified	Unclassified	Healthy	2.51	0.00
Unclassified Subdivision 5	Unclassified Subdivision 5	Healthy	2.49	0.01
Unclassified Lachnospiraceae	Lachnospiraceae	Healthy	2.47	0.02
Unclassified	Unclassified	Healthy	2.42	0.05
Unclassified Subdivision 5	Unclassified Subdivision 5	Healthy	2.39	0.00
Unclassified Ruminococcaceae	Ruminococcaceae	Healthy	2.37	0.03
Unclassified Clostridiales	Unclassified Clostridiales	Healthy	2.36	0.00
Unclassified Clostridiales	Unclassified Clostridiales	Healthy	2.35	0.04
Unclassified Clostridiales	Unclassified Clostridiales	Healthy	2.31	0.01
Unclassified Lachnospiraceae	Lachnospiraceae	Healthy	2.31	0.02
Unclassified Lachnospiraceae	Lachnospiraceae	Healthy	2.31	0.02
Ruminococcus	Ruminococcaceae	Healthy	2.31	0.01
Unclassified Subdivision 5	Unclassified Subdivision 5	Healthy	2.30	0.02
Fibrobacter	Fibrobacteriaceae	Healthy	2.29	0.02
Unclassified Ruminococcaceae	Ruminococcaceae	Healthy	2.28	0.03
Unclassified Proteobacteria	Unclassified Proteobacteria	Healthy	2.28	0.01

Unclassified Firmicutes	Unclassified Firmicutes	Healthy	2.28	0.03
Unclassified Veillonellaceae	Veillonellaceae	Healthy	2.28	0.02
Sporobacter	Ruminococcaceae	Healthy	2.26	0.01
Unclassified Sphingobacteriales	Unclassified Sphingobacteriales	Healthy	2.25	0.02
Unclassified Bacteriodetes	Unclassified Bacteriodetes	Healthy	2.25	0.03
Blautia	Lachnospiraceae	Healthy	2.24	0.01
Unclassified	Unclassified	Healthy	2.23	0.00
Unclassified Clostridiales	Unclassified Clostridiales	Healthy	2.22	0.03
Unclassified Firmicutes	Unclassified Firmicutes	Healthy	2.20	0.03
Unclassified	Unclassified	Healthy	2.19	0.01
Unclassified	Unclassified	Healthy	2.18	0.00
Unclassified Lachnospiraceae	Lachnospiraceae	Healthy	2.18	0.01
Unclassified	Unclassified	Healthy	2.17	0.00
Acetivivbrio	Ruminococcaceae	Healthy	2.17	0.02
Unclassified Subdivision 5	Unclassified Subdivision 5	Healthy	2.15	0.00
Unclassified Bacteriodetes	Unclassified Bacteriodetes	Healthy	2.15	0.03
Unclassified Clostridiales	Unclassified Clostridiales	Healthy	2.14	0.02
Unclassified Firmicutes	Unclassified Firmicutes	Healthy	2.12	0.01
Unclassified Clostridiaceae	Clostridiaceae	Healthy	2.12	0.04
Acetivibrio	Ruminococcaceae	Healthy	2.11	0.02
Acetivibrio	Ruminococcaceae	Healthy	2.10	0.02
Faecalibacterium	Ruminococcaceae	Healthy	2.10	0.02
Unclassified	Unclassified	Healthy	2.09	0.02
Unclassified	Unclassified	Healthy	2.08	0.01
Unclassified	Unclassified	Healthy	2.08	0.03
Unclassified Lachnospiraceae	Lachnospiraceae	Healthy	2.08	0.02
Unclassified	Unclassified	Healthy	2.08	0.01
Unclassified Ruminococcaceae	Ruminococcaceae	Healthy	2.07	0.01
Unclassified Subdivision 5	Unclassified Subdivision 5	Healthy	2.06	0.00
Unclassified	Unclassified	Healthy	2.06	0.02
Unclassified Coriobacteriaceae	Coriobacteriaceae	Healthy	2.05	0.03
Acetivibrio	Ruminococcaceae	Healthy	2.05	0.02
Unclassified Ruminococcaceae	Ruminococcaceae	Healthy	2.04	0.02
Unclassified Lachnospiraceae	Lachnospiraceae	Healthy	2.04	0.03
Unclassified Clostridiales	Unclassified Clostridiales	Healthy	2.04	0.03
Unclassified Clostridiales	Unclassified Clostridiales	Healthy	2.04	0.02
Unclassified Lachnospiraceae	Lachnospiraceae	Healthy	2.04	0.03
Unclassified Ruminococcaceae	Ruminococcaceae	Healthy	2.04	0.02
Unclassified	Unclassified	Healthy	2.04	0.02
Unclassified Lachnospiraceae	Lachnospiraceae	Healthy	2.03	0.01
Unclassified Ruminococcaceae	Ruminococcaceae	Healthy	2.03	0.03
Anaeroplasma	Anaeropalsmaceae	Healthy	2.02	0.03
Unclassified Ruminococcaceae	Ruminococcaceae	Healthy	2.02	0.02
Unclassified Erysopelotrichaceae	Erysopelotrichaceae	Healthy	2.00	0.04
Megasphaeral	Veillonellaceae	Diarrhea	3.53	0.02
Phascolarctobacterium	Acidaminococcaceae	Diarrhea	3.30	0.01
Clostridium XIVb	Lachnospiraceae	Diarrhea	3.25	0.00
Clostridium XVIII	Erysopelotrichaceae	Diarrhea	3.15	0.00
Butyricicoccus	Ruminococcaceae	Diarrhea	3.01	0.00

Megasphaeral	Veillonellaceae	Diarrhea	3.53	0.02
Phascolarctobacterium	Acidaminococcaceae	Diarrhea	3.30	0.01
Clostridium XIVb	Lachnospiraceae	Diarrhea	3.25	0.00
Clostridium XVIII	Erysopelotrichaceae	Diarrhea	3.15	0.00
Butyricicoccus	Ruminococcaceae	Diarrhea	3.01	0.00
Parabacteroides	Porphyromonadaceae	Diarrhea	3.00	0.02
Butyricicoccus	Ruminococcaceae	Diarrhea	2.94	0.02
Streptococcus	Streptococcaceae	Diarrhea	2.88	0.01
Oscillibacter	Ruminococcaceae	Diarrhea	2.82	0.04
Blautia	Lachnospiraceae	Diarrhea	2.77	0.03
Unclassified Ruminococcaceae	Ruminococcaceae	Diarrhea	2.74	0.01
Unclassified Lachnospiraceae	Lachnospiraceae	Diarrhea	2.62	0.04
Alistipes	Rikenellaceae	Diarrhea	2.53	0.01
Unclassified Lachnospiraceae	Lachnospiraceae	Diarrhea	2.51	0.04
Streptococcus	Streptococcaceae	Diarrhea	2.21	0.04
Unclassified Firmicutes	Unclassified Firmicutes	Diarrhea	2.20	0.04
Unclassified Lachnospiraceae	Lachnospiraceae	Diarrhea	2.10	0.04
Sporobacter	Ruminococcaceae	Diarrhea	2.01	0.01
Alistipes	Rikionellaceae	Diarrhea	2.01	0.00

T1: 1-14 days of age, T2: 15-28 days of age
